# Surgical Clinic Nurses’ Empathy Levels and Attitudes Toward Patients with Disabilities: A Cross-Sectional Study

**DOI:** 10.3390/healthcare14121604

**Published:** 2026-06-06

**Authors:** Harun Ünal, Gültaç Erfidan, Ömer Kümet, Murat Koç, Zeynep Karaman Özlü

**Affiliations:** 1 Faculty of Health Sciences, Van Yuzuncu Yil University, Van 65000, Turkey; 2 Health Technician Department, Van Health Services Vocational School, Van Yuzuncu Yil University, Van 65000, Turkey; gultacerfidan@yyu.edu.tr; 3 Cardiology Department, Van Educational and Research Hospital Health Science University, Van 65000, Turkey; omerkumet@hotmail.com; 4 Pendik State Hospital, İstanbul 34000, Turkey; mrtkoc49@gmail.com; 5 Department of Surgical Nursing, Faculty of Nursing, Ataturk University, Erzurum 25000, Turkey; zynp_krmnzl@hotmail.com

**Keywords:** surgical nursing, cross-sectional studies, patient care, empathy, attitude to persons with disabilities

## Abstract

**Background:** The objective of the current study is to identify the empathy levels and attitudes of nurses who work in surgical clinics toward patients with disabilities and to investigate the correlation between these two variables. **Methods:** This cross-sectional, correlational-predictive study included 305 nurses working in the surgical clinics of a Training and Research Hospital affiliated with a Health Sciences University in Türkiye. The research data were collected using a Sociodemographic Information Form, the Jefferson Empathy Scale (JES), and the Disability Attitudes in Health Care Scale (DAHC) between February and April 2026. Descriptive statistics, Pearson’s correlation analysis, POMP (%) score graph, eta-squared (η^2^) effect size, independent samples t-test, one-way analysis of variance (ANOVA), and hierarchical multiple linear regression analysis were used to evaluate the data. **Results**: Participants scored an average of 102.8 points on the JES and 55.4 points on the DAHC. This value falls within Jefferson’s “moderately high empathy” range (88.6–105.7), and the mean attitude score was also found to increase positively. A positive, moderate, and statistically significant correlation was found between the JES and the DAHC (r = 0.520, *p* < 0.001). In the regression analysis, the explanatory power increased from 12.8% in Model 1 to 18.8% in Model 2 and to 35% in Model 3 (ΔR^2^ = 0.160). Considering the relative importance order of the independent variables in the final model of the regression analysis, it is understood that the strongest predictor of empathy was the DAHC (β = 0.430), followed by professional experience (β = 0.149) and education level (β = −0.145). **Conclusions**: The present study found that attitude, education level, and professional experience were the main factors affecting the empathy level. A positive correlation was identified between empathy and attitude. Hence, the accessibility of patients with disabilities to intensive and stressful clinics such as surgical clinics, the organization of in-service training, and the integration of multidisciplinary teamwork can create equal opportunities in health care for patients with disabilities.

## 1. Introduction

The concept of disability refers to the loss of physical, mental, emotional, and social abilities to varying degrees due to congenital or acquired causes, leading to difficulties in adapting to social life and meeting daily needs and requiring protection, care, rehabilitation, counseling, and support services [1]. According to the data from the World Health Organization, approximately 16% of the world’s population, or 1.3 billion people, have some type of disability. Additionally, 190 million individuals face serious difficulties in daily living functions [2]. According to the Old Age Population Statistics Bulletin, there are a total of 2,511,950 Patients with Disabilities in Türkiye; of these, 1,414,643 are male, and 1,097,307 are female [3]. When individuals with chronic diseases are also included, about 8.5 million individuals in Türkiye experience limitations in diverse areas and face numerous challenges in social life [4]. Inequalities that individuals with disabilities encounter in accessing healthcare services originate not only from existing barriers but also from healthcare professionals’ beliefs and attitudes regarding social minorities and different groups [5]. Thus, some studies have demonstrated that patients with disabilities are exposed to more inequalities in accessing healthcare services than individuals without disabilities [6,7,8] and that healthcare providers prefer individuals without disabilities when delivering healthcare services [5]. Negative attitudes toward patients with disabilities are widespread among healthcare professionals [9,10]. It is stressed that these negative approaches and attitudes increase inequalities in access to healthcare services [11]. In this regard, a better understanding of differences toward individuals with disabilities is essential to reduce inequalities in access to healthcare services, improve interventions, increase accessibility, and strengthen awareness campaigns [12].

Empathy is another critical issue in healthcare. Empathy represents a basic element of the relationship between nurses and patients and is addressed as an important subject in the literature on nursing care [13]. Nurses, who constitute a significant portion of healthcare professionals, provide care to patients with disabilities in all segments of society and play an essential role in their physical, mental, and social empowerment [14]. Nurses must adopt an empathetic approach to accurately understand patients’ needs and achieve more positive health outcomes [15]. Numerous studies have shown that healthcare professionals’ levels of empathy correlate with the quality of patient care; high empathy levels both increase patient satisfaction and enhance health outcomes [16]. While providing basic care to patients in the pre- and post-operative periods, surgical clinic nurses may also witness intense emotions such as fear, anxiety, and hope, and changes in the quality of life of patients, particularly those undergoing major surgery, can challenge surgical clinic nurses’ empathy and compassion [17]. Whereas the number of studies focusing on the relationship between disability and empathy among physicians, medical and nursing students is increasing [18,19,20], the situation of surgical clinic nurses still constitutes a gap in the literature. Considering that high stress and burnout can suppress empathy among surgical clinic nurses [21], patients with disabilities who undergo treatment in these units are at increased risk of exclusion or inadequate care.

For this reason, it is of critical importance to investigate how surgical clinic nurses’ levels of empathy interact with their attitudes toward individuals with disabilities in order to develop strategies aimed at improving the quality of perioperative care. Accordingly, the objective of the current study is to identify the empathy levels of nurses working in surgical clinics and their attitudes toward patients with disabilities and to examine the relationship between the two variables in question.

Hypotheses

**H1.**  *Surgical clinic nurses’ attitudes toward patients with disabilities are above average*.

**H2.**  *Surgical clinic nurses’ levels of empathy are above average.*

**H3.**  *Surgical clinic nurses’ levels of empathy correlate positively with their attitudes toward patients with disabilities.*

## 2. Materials and Methods

### 2.1. Study Design

The present research was conducted using a cross-sectional, correlational-predictive design to examine the predictive power of surgical clinic nurses’ attitudes toward patients with disabilities.

### 2.2. Population and Sample

The population of this study comprised nurses working in surgical clinics of a training and research hospital in eastern Türkiye. In the sample selection process of the study, convenience sampling, one of the non-probability sampling methods, was employed due to time and logistical constraints associated with reaching the entire population; participation in the study was conducted on a voluntary basis. The sample size of the study was determined by a priori power analysis, and analyses were conducted using the program G*Power (v3.1.9.7). To ensure the power to explain the total variance of the multivariate structure formed by the control variables and principal predictors in the regression model, the sample size was calculated using the “Linear multiple regression: Fixed model, R^2^ deviation from zero” option. The said approach aims to reduce the probability of Type II errors. The total number of predictors was determined to be 21 by adding one continuous variable to the 20 parameters obtained after dummy coding of the categorical variables in the model [22]. In line with this, the minimum sample size required for 21 predictors was calculated to be 226 as a result of the power analysis conducted at f^2^ = 0.15, significance level of α = 0.05, and statistical power of 1 − β = 0.95. Considering possible data losses that may occur due to missing data, erroneous data entry, or exclusion of outliers from the analysis during data collection, approximately 25% was added to the minimum sample size calculated in accordance with methodological recommendations [23]. Thus, the target sample size was updated to 301 participants, resulting in a total of 305 study participants. The inclusion criteria were determined as follows: being a nurse working in surgical clinics of a hospital, being aged between 18 and 65 years, and volunteering to participate in the study. Nurses who were not open to communication or who had a physical or psychological condition that would prevent their participation in the research were excluded.

### 2.3. Instruments

#### 2.3.1. Sociodemographic Information Form

The form in question consists of 9 questions about participants’ socio-demographic, occupational, and disability-related characteristics.

#### 2.3.2. Jefferson Empathy Scale (JES)

The Jefferson Empathy Scale (JES) was developed by Hojat et al. (1985) [24], and Yanık and Saygılı conducted a validity and reliability study for the scale in Turkish [25]. Cronbach’s alpha coefficient of the original JES scale was determined to be 0.77. This scale was developed with the objective of evaluating participants’ current experiences and measuring individuals’ self-reported empathic orientation. The JES is a Likert scale (1 = Strongly disagree, 7 = Strongly agree), comprising 20 items and 3 dimensions. The sub-dimensions of the JES include perspective taking (PT), compassionate care (CC), and standing in the patient’s shoes (SPS), and items 1, 3, 6, 7, 8, 11, 12, 14, 18, and 19 are reverse-scored. A minimum score on the scale is 20, and a maximum score is 140; empathic adaptation increases with the increased scale score. This study found the following Cronbach’s alpha internal consistency coefficients: 0.862 for the PT subscale, 0.763 for the CC subscale, 0.705 for the SPS subscale, and 0.882 for the overall scale.

#### 2.3.3. Disability Attitudes in Health Care Scale (DAHC)

The said scale was developed by Chadd and Pangilinan (2011) [26], and Şahin and Gedik (2020) adapted the DAHC into Turkish [27]. The DAHC is a 15-item Likert scale. Items 2, 5, 6, 7, 8, 12, 13, 14, and 15 are reverse-scored, and the total score is computed by summing the answers to all items. A high total score indicates a positive attitude toward patients with disabilities. The DAHC has two subscales: “Burden” and “Inclusion.” The Burden subscale reflects negative attitudes toward patients with disabilities and the tendency to regard these individuals as a burden in healthcare services. A higher score on this subscale indicates that healthcare professionals do not perceive patients with disabilities as a burden. The Inclusion subscale measures whether there is an inclusive approach toward individuals with disabilities in healthcare [28]. The present study found the following Cronbach’s alpha internal consistency coefficients: 0.733 for the Burden subscale, 0.769 for the Inclusion subscale, and 0.802 for the overall scale.

### 2.4. Data Collection

The current research was carried out between February and April 2026. Data were collected from nurses working in surgical clinics through face-to-face methods and online by sharing the scale link via email and WhatsApp groups. It took about 15–20 min to evaluate and complete the scales. Before the study, informed consent was obtained from participants who participated face-to-face in writing and from participants who participated online electronically, stressing anonymity and confidentiality (e.g., via a checkbox in Google Docs).

### 2.5. Data Analysis

The research data were analyzed using the IBM SPSS 26 software package. Within the scope of descriptive statistics, frequency (n), percentage (%), arithmetic mean and standard deviation ($\bar{X}$ ± sd), median, mode, and minimum-maximum values were calculated [23]. Pearson’s correlation analysis examined relationships between the variables [29]. To make the scale scores comparable, raw scores were standardized using the “Percentage of Maximum Possible” (POMP) method and converted to a range of 0–100. The scales’ internal consistency was evaluated using Cronbach’s alpha (α) coefficient, and values ≥0.70 were accepted [30]. A confidence interval of 95% was adopted in all analyses, and *p* < 0.05 was accepted as the criterion for statistical significance. The eta-squared (η^2^) effect size was calculated for significant differences; 0.01 was interpreted as a small, 0.06 as a medium, and 0.14 as a large effect [31,32]. The independent samples t-test and one-way analysis of variance (ANOVA) compared the groups. A hierarchical regression model was established in the study.

## 3. Results

Of the surgical clinic nurses participating in the study, 53.1% were aged between 29 and 39 years, 50.2% were male (n = 153), 64.6% were married, 55.7% held a bachelor’s degree, 81.6% did not have a patients with a disability in their family, 35.1% had 6–10 years of professional experience, 51.1% had not previously cared for a patients with a disability, and 85.6% had not received in-service training (Table 1).

Participants scored an average of 102.8 points on the Jefferson Empathy Scale. This value is within Jefferson’s (88.6–105.7) “above-average empathy” range, confirming that the POMP score corresponds to 69% of the scale (above-average). Participants scored an average of 55.4 points on the Disability Attitudes in Health Care Scale. The value above corresponds to 67.3% of the scale (Table 2).

**Figure 1:**
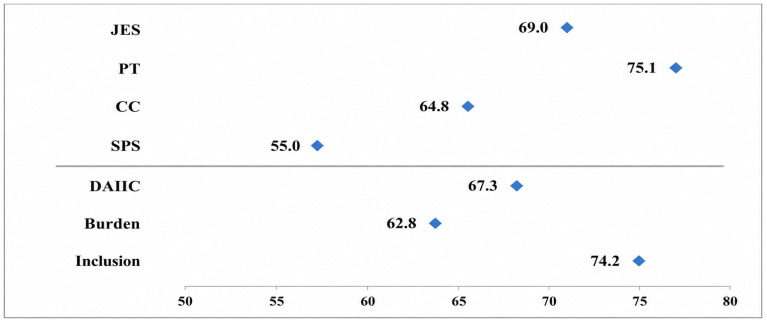
Cleveland dot plot of the scale POMP (%) scores.

Although participants generally demonstrated relatively high empathy levels (JES, POMP = 69%), Perspective Taking (PT) had the highest POMP score among the subdimensions (75.1%). This was followed by Compassionate Care (CC, 64.8%), whereas the lowest POMP score was observed for Standing in the Patient’s Shoes (SPS, 55%) (Figure 1). The mean Disability Attitudes in Health Care Scale (DAHC) score among surgical nurses was determined to be 67.3% (Figure 1).

There is a positive and statistically significant correlation between the sub-dimensions of the JES, PT (r = 0.860), CC (r = 0.738), and SPS (r = 0.384) (*p* < 0.001). Whereas there is a strong correlation with PT and CC of the JES (r > 0.70), this correlation with SPS is weak (r < 0.40). There is a positive, statistically significant, and strong correlation between the sub-dimensions of the DAHC, Burden (r = 0.898) and Inclusion (r = 0.780) (*p* < 0.001). However, the correlation between the sub-dimensions decreases to a moderate level (r = 0.424). To ensure comparability among scale scores with different numbers of items and score ranges, raw scores were standardized using the “Percentage of Maximum Possible” (POMP) method and transformed to a 0–100 scale. These findings confirm the POMP scores in Figure 1. There is a positive, moderate, and statistically significant correlation between the JES and the DAHC (r = 0.520, *p* < 0.001) (Table 3).

As seen in Table 4, a statistically significant difference in the JES was determined according to the age and education variables. Statistically significant differences were found in both the DAHC and JES according to marital status, the presence of a patient with a disability in the family, length of professional experience, previous experience in caring for patients with disabilities, and status of receiving in-service training on disability (*p* < 0.001) (Table 4).

A hierarchical multiple regression analysis was conducted to examine the predictors of empathy among surgical clinic nurses. The model was structured in three sequential blocks to evaluate the stepwise contribution of variables to empathy levels. This hierarchical approach allows the examination of the unique and incremental contribution of different variable groups. Accordingly, sociodemographic characteristics were entered in the first block, occupational and experiential variables in the second block, and attitudes toward persons with disabilities (DAHC) were included in the final block as the main independent variable. Prior to the analysis, regression assumptions were examined. Normality of residuals, linearity between variables, and homoscedasticity were assessed through diagnostic plots, and no serious violations were observed. Multicollinearity was evaluated using the Variance Inflation Factor (VIF) values, and no multicollinearity problem was detected. The Durbin–Watson statistic indicated no evidence of autocorrelation among residuals.

Model 1: First, sociodemographic characteristics were entered into the model, and it was found that at least one variable significantly predicted the JES (F_(4;300)_ = 12.2, *p* < 0.001). It was understood that it explained 14.0% of the variance in the JES (R^2^ = 0.140; Adjusted R^2^ = 0.128). In the first model, marital status, education level, and the presence of a patient with a disability in the family were found to significantly predict empathy (Table 5).

Model 2: Participants’ occupational and experiential characteristics were included along with their sociodemographic characteristics in Model 2, and it was found that at least one variable significantly predicted the JES (F_(7;297)_ = 11.1, *p* < 0.001). These variables added in Model 2 increased the proportion of variance explained to 20.7% (R^2^ = 0.207; Adjusted R^2^ = 0.188). This increase was statistically significant, and the newly added variables explained 6.7% of the variance alone (ΔR^2^ = 0.067, F^d^ = 8.340, *p*^d^ < 0.001). Regarding marital status, single individuals had, on average, 4.67 units lower empathy scores than married individuals. Participants who had a patient with a disability in their family had 5.08 units higher JES scores than participants without a patient with a disability in their family. Participants with a previous experience in caring for patients with disabilities had, on average, 4.18 units higher empathy scores than those without such an experience. Individuals who had received in-service training on disability had, on average, 6.02 units higher JES scores than those who had not received such education. Furthermore, professional experience and education level are the other significant predictors of this model (Table 5).

Model 3: The main independent variable, DAHC, was added to the final model, increasing the total variance to 36.7% (R^2^ = 0.367; Adjusted R^2^ = 0.350). This increase was statistically significant, and the newly added variables explained 16.0% of the variance alone (ΔR^2^ = 0.160, F^d^ = 75.1, *p*^d^ < 0.001). Education level and professional experience continued to be significant predictors of the JES in the final model. High school and associate degree graduates had, on average, 5.1 units lower JES scores than those with bachelor’s degrees or higher. Participants with 11 years or more of professional experience had, on average, 4.64 higher mean empathy scores than those with less than 10 years of professional experience. Attitudes toward individuals with disabilities (DAHC), which were added to the model in the final step, were found to be a positive and significant predictor of empathy (B = 0.72, 95% CI [0.56, 0.89], β = 0.430, t = 8.663, *p* = 0.000). It was determined that each 1-unit increase in the DAHC score significantly increased the empathy score by 0.72 units (Table 5).

**Figure 2:**
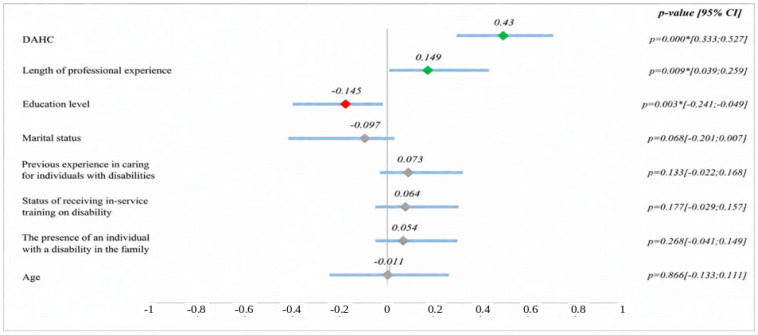
Forest plot for the final model and predictors of the JES. Diamonds and the values on them represent the standardized beta coefficient (β), horizontal light blue lines represent the 95% confidence intervals [95% CI] for the standardized beta coefficient determined using the Delta method, the vertical dashed line represents the zero reference line, red diamonds represent negative effects, green diamonds represent positive effects, and gray diamonds represent no effect. * *p* < 0.001.

The forest plot visually displays the factors affecting the JES scores. The DAHC is located on the far right of the plot and is the strongest positive predictor (β = 0.43), followed by the length of professional experience (β = 0.149). Education level is the third negative predictor of the JES (β = −0.145) (Figure 2).

## 4. Discussion

The current study evaluated surgical clinic nurses’ empathy levels and their attitudes toward patients with disabilities. To our knowledge, the present research is the first cross-sectional and descriptive study in the literature, investigating the empathy levels and attitudes of nurses who work in surgical clinics toward patients with disabilities. The study found that participants’ mean scores for empathy and attitudes toward individuals with disabilities were moderate-high. Furthermore, there was a positive and moderately significant correlation between empathy and attitude. Attitude, educational level, and professional experience were the main factors associated with empathy levels. The rate of participants who had a patient with a disability in their family was determined to be 18.4% in this study. It is reported that the prevalence of disability in Türkiye ranges from approximately 6.9% to 12.29% depending on the definitions and criteria used, and that this rate can reach 18% in more comprehensive evaluations [33]. Based on the data presented above, it is understood that patients with disabilities are quite widespread in society and that it is a multidimensional phenomenon impacting not only individuals but also their families. The presence of a patient with a disability in the family can lead to increased care needs, emotional burden, and challenges, such as changes in daily life routines.

The study determined that surgical clinic nurses’ attitudes toward patients with disabilities ($\bar{X}$ = 55.4) were above average. The study conducted by Apaydın and Barış (2021) with healthcare professionals found that their attitudes toward patients with disabilities were positive [34]. Likewise, the study carried out by Gültepe (2020) on nurses and midwives revealed that participants displayed moderately positive attitudes toward patients with disabilities [35]. Yıldırım Sarı et al. (2010) reported that participants had positive attitudes toward patients with disabilities [36]. This may be related to the understanding of respect for human dignity and providing equal care, which are among the ethical principles of the nursing profession. It can be stated that the ethical principles in question support the development of accepting and positive attitudes toward patients with disabilities. However, some findings in the literature indicate negative attitudes toward patients with disabilities. These negative attitudes have been reported to affect the success and failure of patients with disabilities and prevent their participation in working life [37]. It is thought that participants’ sociodemographic characteristics and professional experience may be associated with different findings in the literature. Accordingly, surgical clinic nurses’ attitudes toward patients with disabilities were found to be above average, and hypothesis H1 was accepted.

The study determined that surgical clinic nurses’ empathy levels ($\bar{X}$ = 102.8) were above average. This finding is similar to studies in the literature. Özdemir and Kaplan (2024) reported that nurses’ empathy levels were above average [15]. Likewise, Şahin et al. (2018) also detected high empathy levels among nurses [38]. Additionally, Sedaghati Kesbakhi et al. (2017) found that oncology nurses’ empathy levels were above average [39]. The nursing profession focuses on individuals’ needs with a holistic approach and bases empathy on enhancing the quality of care. Hence, empathy helps nurses understand individuals’ feelings and thoughts, which enables the care process to be carried out more effectively and sensitively [40]. However, despite cognitively strong levels of empathy among nurses, high workload, insufficient staffing levels, long working hours, and burnout may be negatively associated with empathetic care behaviors. In this regard, surgical clinic nurses’ empathy levels were found to be above average, and hypothesis H2 was accepted.

The correlation analysis performed in this study found a positive, moderate, and statistically significant correlation between empathy and attitude. The study conducted by Kim and Kwon (2017) with nurses revealed a moderate-to-high positive relationship between participants’ empathy levels and attitudes [41]. The study performed by Yang et al. (2016) on nursing students identified a low-level positive relationship between participants’ empathy and attitude levels [42]. In nursing care, empathy is addressed as the ability to evaluate an individual’s experiences from their own perspective. In this respect, attitude can be considered a cognitive threshold defining the boundaries of the emotional bond that the caregiver will establish. When this threshold is not exceeded, the reflection of an empathic approach in caring behaviors and the holistic fulfillment of the individual’s needs may also become more challenging [43]. Consequently, a significant correlation was identified between surgical clinic nurses’ empathy levels and their attitudes toward patients with disabilities, and hypothesis H3 was accepted.

The regression analysis found that the empathy scores of single individuals were 4.67 units lower on average compared to married individuals; participants who had a patient with a disability in their family had empathy scores 5.08 units higher on average than participants who had no patient with a disability in their family; participants with a previous experience in caring for individuals with disabilities had empathy scores 4.18 units higher on average than those without such an experience; and participants who had received in-service training on disability had empathy scores 6.02 units higher on average than those who had not undergone such training. Although there is insufficient evidence in the literature for the relationship between marital status and attitudes toward patients with disabilities, some studies have demonstrated that individuals who have a family member with a disability [44,45], have an experience in caring for patients with disabilities [46], and who have received training on patients with disabilities [47,48] develop more positive attitudes. These factors are primarily associated with more positive attitudes, while positive attitudes may be indirectly related to the level of empathy and may play a mediating role in this relationship.

The final model of the regression analysis found that the variables of education level and professional experience did not lose their significance. Graduates with bachelor’s and master’s degrees had empathy scores 5.1 units higher on average than high school and associate degree graduates; participants with 11 years or more of professional experience had empathy scores 4.64 units higher on average than those with less than 10 years of experience. Therefore, it can be said that education and professional experience are associated with higher empathy levels, not only through attitude but also independently of attitude. Studies have demonstrated that healthcare professionals with higher education levels and more professional experience display more positive attitudes toward patients with disabilities [28,35,49,50]. The literature has also stressed that higher education levels and increased professional experience positively affect healthcare professionals’ empathy [51,52]. The fact that education level and professional experience are associated with empathy indicates that empathy cannot be considered solely as an emotional tendency. Empathy can be addressed as a skill shaped by a cognitive dimension that develops over time through professional experience and is structured through education.

The final model determined that attitude was the variable that most strongly and significantly associated with the level of empathy in a positive direction. The study conducted by Hamed et al. (2025) on psychiatry and mental health nurses revealed that attitude was positively associated with the level of empathy [20]. The study performed by Yang et al. (2016) on nursing students found that attitude was a factor positively associated with the level of empathy [42]. The said finding suggests that attitude plays a key determinant role in the improvement of empathy levels among nurses and that positive attitudes may be associated with higher empathy levels. Although the final regression model explained approximately 35–36.7% of the variance in empathy levels, a substantial proportion of the variance remained unexplained. This finding suggests that empathy is a multidimensional construct influenced by various individual and psychosocial factors that were not evaluated in the present study. In particular, personality traits, emotional intelligence, psychological resilience, and patient–nurse interaction may have an impact on empathy levels [53]. Therefore, future studies are recommended to examine these variables within broader and more multidimensional models in order to better explain empathy among healthcare professionals.

## 5. Limitations

The fact that the current study draws attention to a topic not adequately addressed in the literature, attitudes toward patients with disabilities in a surgical clinical practice setting, is one of its strengths. Considering the intense and challenging setting of surgical clinics, assessing the empathy levels and attitudes toward patients with disabilities of nurses working in these clinics significantly contributes to developing a patient-centered care approach. Furthermore, it provides a scientific basis for developing inclusive and equitable healthcare services for patients with disabilities, who constitute one of the disadvantaged groups. However, the present study also has some limitations. To the best of our knowledge, this study is among the first to examine the empathy levels and attitudes toward patients with disabilities among nurses working in the surgical clinics of a hospital in Türkiye; therefore, the sample size was limited to 305 participants. Further studies conducted in different countries are needed to strengthen generalizability. Second, this study was conducted only on nurses working in surgical clinics. Furthermore, potential biases associated with self-reported data and the effects of social desirability should be considered among the limitations of the study. Cross-sectional study designs may limit the ability to establish causal inferences between variables. Researching the empathy levels and attitudes toward patients with disabilities of other stakeholders working in the healthcare field (nurses in other clinics, medical secretaries, physicians, caregivers, etc.) is critical to increasing inclusivity and the quality of care in healthcare services.

## 6. Conclusions and Recommendations

This study found that nurses working in surgical clinics had above-average empathy levels and attitudes toward patients with disabilities, and attitudes toward patients with disabilities improved with an increased level of empathy. The study determined that nurses with higher education levels, more than 11 years of professional experience, a patient with a disability in their family, previous experience in caring for patients with disabilities, and having undergone in-service training on disability had significantly higher empathy and attitude scores. In this regard, to enhance the quality of care and the satisfaction of patients with disabilities in stressful units such as surgical units, it is recommended that modules including the rights of patients with disabilities and communication techniques that strengthen empathy skills should be widely implemented, and clinical settings should be made accessible to patients with disabilities. Moreover, to fill the gap in the literature, it is essential to support this subject with comprehensive quantitative and qualitative studies and to examine patient care outcomes.

The study findings highlight the importance of surgical clinic nurses’ empathy levels and attitudes toward patients with disabilities across different healthcare systems. Although the transferability of the findings may vary depending on healthcare policies, cultural values, and working conditions, the results may provide guidance for different cultural contexts due to the universal importance of empathy and inclusive care.

## Figures and Tables

**Table 1:** Participant-Specific Characteristics (n = 305).

Variables	Categories	n	%
Age	18–29	83	27.2
	29–39	162	53.1
	40 and over	60	19.7
Sex	Female	152	49.8
	Male	153	50.2
Marital status	Married	197	64.6
	Single	108	35.4
Education level	High school	29	9.5
	Associate degree	53	17.4
	Bachelor’s degree	170	55.7
	Postgraduate	53	17.4
The presence of a patient with a disability in the family	Yes	56	18.4
	No	249	81.6
Length of professional experience (years) patients	0–5 years	52	17
	6–10 years	107	35.1
	11–15 years	82	26.9
	16 years and more	64	21
Surgical unit of employment	Operating room	57	18.7
	Neurosurgery	24	7.9
	General surgery	46	15.1
	Thoracic surgery	20	6.6
	Ophthalmic surgery	19	6.2
	Obstetrics and gynecology	20	6.6
	Cardiovascular surgery	23	7.5
	Ear, nose, and throat surgery	22	7.2
	Orthopedics	29	9.5
	Plastic surgery	23	7.5
	Urology	22	7.2
Previous experience in caring for patients with disabilities	Yes	149	48.9
	No	156	51.1
Status of receiving in-service training on disability	Yes	44	14.4
	No	261	85.6

n: Number/frequency, %: Percentage.

**Table 2:** Descriptive Findings Regarding the Scales.

	Mean	sd	POMP (%)	Minimum	Maximum	Mode	Median	α
JES	102.8	15.6	69	53	134	114	105	0.882
PT	55.1	10.4	75.1	18	70	64	57	0.862
CC	39.1	7.6	64.8	17	56	37	39	0.763
SPS	8.6	2.8	55	4	14	8	8	0.705
DAHC	55.4	9.3	67.3	27	75	55	55	0.802
Burden	31.6	6.4	62.8	14	45	30	31	0.733
Inclusion	23.8	4.5	74.2	6	30	27	25	0.769

Mean: Mean, sd: Standard deviation, POMP: Percentage of Maximum Possible, α: Cronbach’s alpha reliability coefficient.

**Table 3:** Inter-Scale Correlation.

		JES	PT	CC	SPS	DAHC	Burden	Inclusion
JES	r:	1	0.860 **	0.738 **	0.384 **	0.520 **	0.354 **	0.565 **
PT	r:		1	0.331 **	0.182 *	0.436 **	0.230 **	0.569 **
CC	r:			1	0.175 **	0.370 **	0.329 **	0.294 **
SPS	r:				1	0.279 **	0.233 **	0.243 **
DAHC	r:					1	0.898 **	0.780 **
Burden	r:						1	0.424 **

r: Pearson correlation coefficient, ** *p* < 0.001, * *p* < 0.05.

**Table 4:** Comparison of the Scale Scores According to Variables.

		DAHC			JES		
		$\bar{X}$ ± sd.	F/t (η^2^)	*p*	$\bar{X}$ ± sd.	F/t (η^2^)	*p*
Age	18–29 (1)	54 ± 9.9	1.48	0.229	96.9 ± 19	8.496	0.000 *
	29–39 (2)	55.8 ± 9.2			103.9 ± 13.6	(0.063)	2 > 1 **
	40 and over (2)	56.5 ± 8.3			107.9 ± 12.9		
Sex	Female	55.7 ± 8.8	0.478	0.633	103.5 ± 14.5	0.767	0.444
	Male	55.2 ± 9.7			102.1 ± 16.6		
Marital status	Married	56.5 ± 8.8	2.831	0.005	105.7 ± 13.4	4.528	0.000 *
	Single	53.4 ± 9.7	(0.026)		97.5 ± 17.8	(0.063)	
Education level	High school (1)	54 ± 9.9	1.501	0.214	96.6 ± 17	7.26	0.000 *
	Associate degree (1)	54.7 ± 10.2			96.7 ± 17.8	(0.073)	2 > 1 **
	Bachelor’s degree (2)	55.2 ± 8.8			106.8 ± 14.3		
	Postgraduate (2)	57.8 ± 9.3			109.1 ± 13.2		
The presence of a patient with a disability in the family	Yes	59.9 ± 8.4	4.075	0.000 *	110.1 ± 14	3.964	0.000 *
	No	54.4 ± 9.2	(0.052)		101.2 ± 15.5	(0.049)	
Length of professional experience (years)	0–5 years (1)	53.4 ± 10.1	2.92	0.034 *	94.1 ± 21.1	10.312	0.000 *
	6–10 years (1)	54.3 ± 9.8	(0.028)	2 > 1 **	99.7 ± 14.4	(0.107)	2 > 1 **
	11–15 years (2)	57.4 ± 8.5			108.5 ± 11.6		
	16 years + (2)	56.5 ± 8.1			106.2 ± 12.8		
Surgical unit of employment	Operating room	54.3 ± 7.5	0.793	0.636	105.4 ± 13.1	1.051	0.401
	Neurosurgery	57 ± 11.7			100.3 ± 17.9		
	General surgery	55.1 ± 8.6			100.3 ± 18		
	Thoracic surgery	55.3 ± 7.4			105.5 ± 14.5		
	Ophthalmic surgery	55.1 ± 9			105.9 ± 14.8		
	Obstetrics and gynecology	56.9 ± 12			101.8 ± 14		
	Cardiovascular surgery	54.6 ± 10.2			99.1 ± 18.1		
	Orthopedics	55.8 ± 10.8			101.8 ± 17.9		
	Plastic surgery	53.2 ± 8.8			101 ± 15.6		
	Urology	59.5 ± 8.9			109 ± 13.4		
	Ear, nose, and throat surgery	55.3 ± 8.9			100.8 ± 11.2		
Previous experience in caring for patients with disabilities	Yes	57.3 ± 8.9	3.463	0.001 *	106.1 ± 13.4	3.628	0.000 *
	No	53.7 ± 9.3	(0.038)		99.7 ± 16.8	(0.042)	
Status of receiving in-service training on disability	Yes	60 ± 9.1	3.629	0.000 *	109.6 ± 13.1	3.169	0.002 *
	No	54.7 ± 9.1	(0.042)		101.7 ± 15.6	(0.032)	

$\bar{X}$ ± sd.: Mean plus/minus standard deviation, F: ANOVA test, t: Independent samples *t*-test, η^2^: Eta-squared effect size, ** Post hoc intercategory comparisons * *p* < 0.05.

**Table 5:** Hierarchical Multiple Regression Analysis Results for the Independent Variables Affecting the JES.

	B	SE.	LL	UL	β	t	*p*.
**Model 1: Sociodemographic characteristics** (F: 12.2, df1: 4, df2: 300, *p* < 0.001)							
(Constant)	105.9	1.25	103.5	108.4		84.65	0.000
Age	−2.84	2.18	−7.13	1.46	−0.081	−1.299	0.195
Marital status (RC: Married, 1: Single)	−5.67	1.96	−9.53	−1.82	−0.174	−2.896	0.004 *
Education level (RC: Bachelor’s degree and above, 1: High school and Associate degree)	−5.82	1.98	−9.72	−1.93	−0.166	−2.94	0.004 *
The presence of a patient with a disability in the family (RC: No, 1: Yes)	6.72	2.19	2.41	11.03	0.167	3.07	0.002 *
R: 0.374, R^2^: 0.140, R^a^: 0.128, ΔR^2^: 0.140, F^d^: 12.2, *p*^d^ < 0.001, VIF: 1.04–1.36							
**Model 2: Occupational and experiential characteristics** (F: 11.1, df1: 7, df2: 297, *p* < 0.001)							
(Constant)	98.7	1.99	94.8	102.6		49.67	0.000
Age	0.53	2.41	−4.21	5.26	0.015	0.218	0.827
Marital status (RC: Married, 1: Single)	−4.67	1.9	−8.41	−0.93	−0.144	−2.455	0.015 *
Education level (RC: Bachelor’s degree and above, 1: High school and Associate degree)	−4.94	1.92	−8.72	−1.16	−0.141	−2.57	0.011 *
The presence of a patient with a disability in the family (RC: No, 1: Yes)	5.08	2.15	0.84	9.32	0.126	2.357	0.019 *
Length of professional experience (RC: 0–10, 1:11+)	6.5	1.95	2.66	10.33	0.209	3.335	0.001 *
Previous experience in caring for patients with disabilities (RC: No, 1: Yes)	4.18	1.66	0.92	7.44	0.134	2.521	0.012 *
Status of receiving in-service training on disability (RC: No, 1: Yes)	6.02	2.32	1.45	10.58	0.136	2.594	0.010 *
R: 0.454, R^2^: 0.207, R^a^: 0.188, ΔR^2^: 0.067, F^d^: 8.340, *p*^d^ < 0.001, VIF: 1.28–1.74							
**Model 3: DAHC** (F: 21.5, df1: 8, df2: 296, *p* < 0.001)							
(Constant)	61.1	4.69	51.9	70.3		13.04	0.000
Age	−0.38	2.16	−4.62	3.86	−0.011	−0.177	0.86
Marital status	−3.14	1.71	−6.51	0.23	−0.097	−1.835	0.068
Education level (RC: Bachelor’s degree and above, 1: High school and Associate degree)	−5.1	1.72	−8.48	−1.71	−0.145	−2.964	0.003 *
The presence of a patient with a disability in the family	2.17	1.96	−1.68	6.02	0.054	1.111	0.268
Length of professional experience (RC: 0–10, 1:11+)	4.64	1.76	1.19	8.1	0.149	2.643	0.009 *
Previous experience in caring for patients with disabilities	2.26	1.5	−0.69	5.21	0.073	1.505	0.133
Status of receiving in-service training on disability	2.85	2.11	−1.3	7.00	0.064	1.352	0.177
DAHC	0.72	0.08	0.56	0.89	0.430	8.663	0.000 *
R: 0.606, R^2^: 0.367, R^a^: 0.350, ΔR^2^: 0.160, F^d^: 75.1, *p*^d^ < 0.001, VIF: 1.60–1.78, DW: 1.847							

B: Unstandardized beta, SE: Standard error for B; LL-UL: Lower and upper limits at 95% confidence interval for B. β: Standardized beta coefficient, t: Independent samples *t*-test. R^a^: Adjusted R^2^, ΔR^2^: R-squared change, F^d^: F-change; *p*^d^: Significance for F-change. F: ANOVA, VIF: Variance Inflation Factor, DW: Durbin-Watson test, RC: Reference category, * *p* < 0.05.

## Data Availability

The data used in this study are available from the corresponding author upon reasonable request.
